# Injectable bioactive glass/sodium alginate hydrogel with immunomodulatory and angiogenic properties for enhanced tendon healing

**DOI:** 10.1002/btm2.10345

**Published:** 2022-06-03

**Authors:** Hongtao Xu, Yanlun Zhu, Jiankun Xu, Wenxue Tong, Shiwen Hu, Yi‐Fan Chen, Shuai Deng, Hao Yao, Jie Li, Chien‐Wei Lee, Hon Fai Chan

**Affiliations:** ^1^ Musculoskeletal Research Laboratory, Department of Orthopedics and Traumatology The Chinese University of Hong Kong Hong Kong SAR China; ^2^ Innovative Orthopaedic Biomaterial and Drug Translational Research Laboratory, Li Ka Shing Institute of Health Sciences The Chinese University of Hong Kong Hong Kong SAR China; ^3^ Department of Orthopedics The First Affiliated Hospital of Nanjing Medical University Nanjing China; ^4^ Institute for Tissue Engineering and Regenerative Medicine, Faculty of Medicine The Chinese University of Hong Kong Hong Kong SAR China; ^5^ Key Laboratory for Regenerative Medicine of the Ministry of Education of China, School of Biomedical Sciences, Faculty of Medicine The Chinese University of Hong Kong Hong Kong SAR China; ^6^ School of Materials Science and Engineering Lanzhou University of Technology Lanzhou China; ^7^ The Ph.D. Program for Translational Medicine, College of Medical Science and Technology Taipei Medical University Taipei Taiwan; ^8^ Graduate Institute of Translational Medicine, College of Medical Science and Technology Taipei Medical University Taipei Taiwan; ^9^ International Ph.D. Program for Translational Science, College of Medical Science and Technology Taipei Medical University Taipei Taiwan; ^10^ Master Program in Clinical Genomics and Proteomics, School of Pharmacy Taipei Medical University Taipei Taiwan; ^11^ Division of Spine Surgery, Department of Orthopedic Surgery, Nanjing Drum Tower Hospital The Affiliated Hospital of Nanjing University Medical School Nanjing China; ^12^ Center for Translational Genomics Research China Medical University Hospital, China Medical University Taichung Taiwan; ^13^ Hong Kong Branch of CAS Center for Excellence in Animal Evolution and Genetics The Chinese University of Hong Kong Hong Kong SAR China; ^14^ Center for Neuromusculoskeletal Restorative Medicine Hong Kong Science Park Hong Kong SAR China

**Keywords:** bioactive glass, hydrogel, immunomodulatory, injectable, tendon healing

## Abstract

Tendon healing is a complex process involving inflammation, proliferation, and remodeling, eventually achieving a state of hypocellularity and hypovascularity. Currently, few treatments can satisfactorily restore the structure and function of native tendon. Bioactive glass (BG) has been shown to possess immunomodulatory and angiogenic properties. In this study, we investigated whether an injectable hydrogel fabricated of BG and sodium alginate (SA) could be applied to enhance tenogenesis following suture repair of injured tendon. We demonstrated that BG/SA hydrogel significantly accelerated tenogenesis without inducing heterotopic ossification based on histological analysis. The therapeutic effect could attribute to increased angiogenesis and M1 to M2 phenotypic switch of macrophages within 7 days post‐surgery. Morphological characterization demonstrated that BG/SA hydrogel partially reverted the pathological changes of Achilles tendon, including increased length and cross‐sectional area (CSA). Finally, biomechanical test showed that BG/SA hydrogel significantly improved ultimate load, failure stress, and tensile modulus of the repaired tendon. In conclusion, administration of an injectable BG/SA hydrogel can be a novel and promising therapeutic approach to augment Achilles tendon healing in conjunction with surgical intervention.

## 1 INTRODUCTION

Promoting tendon healing remains an unsolved challenge in orthopedics. As the largest and strongest tendon in the human body, Achilles tendon is one of the most frequently ruptured tendons, with an incidence of 12–18 per 100,000.^1^, ^2^ Healing of ruptured Achilles tendon is difficult because of its limited potential for regeneration.^3^ In addition, the remaining Achilles tendon tissue often bears appreciable loads, inevitably leading to further progressive damage.^4^, ^5^ Surgical interventions present several advantages, including lower re‐rupture rates, a larger ultimate range of motion, a shorter time to recovery, and better treatment outcomes when compared with conservative medical treatments.^6^, ^7^, ^8^ However, surgical interventions often cannot restore the native composition, structure, and mechanical properties of the Achilles tendon and can result in tendon lengthening and calf muscle atrophy. Patient‐reported outcomes revealed that bulky functional deficits persist in the injured limb relative to the contralateral side even at 2 years post‐surgery following Achilles tendon injury.^9^ Therefore, there is a clinical need to develop advanced therapies for tendon healing.

In recent years, biomaterials, such as implantable biomaterial scaffolds, have been widely used in preclinical studies for tendon repair.^10^, ^11^ The potential for clinical translation of biomaterial scaffolds depends on factors such as suture retention strength, handling characteristics with respect to the intended clinical application, and resistance to infection. Meanwhile, the interaction of host immune cells, in particular macrophages, with implanted biomaterials has been considered the precursor to granulation tissue formation, classical foreign body reaction, and eventual fibrous encapsulation, which have been associated with negative impacts on device functionality and considered the major factor of ultimate failure of such biomaterials.^12^ Bioactive glasses (BG), which are composed of 45% SiO_2_, 24.5% Na_2_O, 24.5% CaO, and 6% P_2_O_5_, are clinically approved biomaterials and represent a promising inorganic material for tissue engineering and regenerative medicine applications due to its immunomodulatory and angiogenic properties.^13^, ^14^, ^15^ Our previous studies have demonstrated that BG polarized macrophages toward M2 phenotype, which in turn stimulated collagen reorganization, endothelial cell homing, and new blood vessel formation to accelerate the skin wound healing process.^15^, ^16^, ^17^ Similar to skin wound healing, tendon healing is a dynamic and complicated process that can be mainly divided into three phases: inflammation, proliferation, and remodeling.^18^ The inflammatory phase, which represents the immediate response to the injury and lay the groundwork for the remaining two phases, plays a pivotal role in tendon healing.^19^ Meanwhile, the hypovascularity of tendon tissue also slows the healing process.^20^ Therefore, BG may be a potential option to augment tendon healing after surgical treatment via immunomodulation and enhancing angiogenesis, which can potentially lead to functional recovery.

Apart from BG, a variety of biomaterials can be utilized as scaffold for tissue engineering, such as collagen,^21^ fibrin,^22^ silk,^23^ and gelatin methacryloyl.^24^, ^25^ These biomaterials present different advantages, such as exhibiting excellent biological properties and tunable mechanical properties. Some of them can be designed to be injectable to fill any shape of the defect and be easily applied using standard arthroscopic techniques^26^, ^27^, ^28^ Injectable biomaterials for therapeutic agent delivery are advantageous because they can: (i) minimize the damaging effects of large muscle retraction; (ii) shorten the surgical operation time; (iii) reduce surgical‐related pain and scar size; (iv) accelerate post‐surgery recovery; and (v) reduce medical cost.^29^ Although BG is fabricated and supplied in an implantable powder form, pure BG powder frequently causes a high pH value at the repair site, which incurs pain to patients.^15^ Therefore, we proposed a simple approach to combine BG with sodium alginate (SA) to generate an injectable hydrogel. Such a configuration will allow BG to slowly dissolve and release ions gradually. This would result in a less alkaline environment and thus alleviate pain when the hydrogel is administered to patients.^30^ Moreover, the ions released, such as calcium, will trigger the crosslinking of alginate and facilitate in situ gelation of the injectable hydrogel without requiring external stimuli (e.g., ultraviolet light). Finally, alginate is biocompatible and clinically approved for medical applications, rendering our therapeutic approach attractive for clinical translation.

This study aimed to investigate the effect of the injectable BG/SA hydrogel for Achilles tendon repair in a rat model. To the best of our knowledge, this study presents the first report regarding the application of BG/SA in Achilles tendon healing. Our study may facilitate the development of novel strategies for the treatment of tendon rupture.

## 2 MATERIALS AND METHODS

### 2.1 Preparation of BA/SA hydrogel

SA powders derived from brown algae (SA, medium viscosity, Sigma, USA) were dissolved in deionized water to produce a 2% (w/v) SA solution. The SA solution was sterilized with a 0.22 μm filter (Millipore). BG powders and gluconic acid lactone (GDL, Sigma, USA) were both sterilized by ultraviolet light before use. The injectable BG/SA hydrogel was prepared according to the procedures described previously.^31^ Briefly, 5 ml of SA solution was loaded into an injector connected to a t‐branch pipe, before 0.1 g BG powders and 0.05 g GDL powders were added into the injector. After mixed and injected, BG/SA formed a hydrogel in 5 min at room temperature.

### 2.2 Animals and experimental designs

The animal experiments were performed with the approval of the Animal Experimentation Ethics Committee of the Chinese University of Hong Kong (Ref. No. 20‐251‐MIS). Seventy‐two Sprague–Dawley (SD) rats (male, 12–14 weeks old, weight: 350–420 g) were used. The rats were divided into four groups: control group (contralateral side of SR and/or SA group), suture repair group (SR group, treated with suture repair using Kessler technique), suture repair + SA implantation group (SA group, treated with suture repair and SA solution injection only), and suture repair + BG/SA implantation group (BG/SA group, treated with suture repair and BG/SA injection). Achilles tendon samples were harvested for histological analysis and morphological characterization on Days 3, 7, 14, 28, and calf muscle–Achilles tendon–calcaneus bone complexes were harvested for biomechanical testing on Day 28.

### 2.3 Surgery

The Achilles tendon surgery was conducted under general anesthesia using ketamine and xylazine. After the surgical site was shaved, an 8‐mm long skin incision was performed directly on the superficial zone of Achilles tendon. Then Achilles tendon was transected using a carbon steel scalpel blade 5 mm from the proximal end of the calcaneal bone insertion. The tendons were then sutured with the Kessler technique, followed by the injection of 20 μl SA solution or BG/SA hydrogel around the repair sites. The skin was then sutured with 4–0 monofilament nylon. Following surgery, all experimental ankles were immobilized using a manufactured syringe to limit the range of motion, which could prevent tendon re‐rupture post‐surgery.^32^ All rats were returned to their cages and fed with a standard diet.

### 2.4 Histological procedures

For histology, tendon tissue samples were fixed in 4% paraformaldehyde for 48 h at room temperature, followed by paraffin embedding and sectioning at 5 μm thickness. For the evaluation of the healing process, sections were stained with hematoxylin and eosin (H&E) and Masson's trichrome. For the evaluation of heterotopic ossification, Alizarin Red staining was also performed. For immunostaining, the following antibodies were used: anti‐Scleraxis (Abcam, SCX, catalog # ab58655, 1:50), anti‐Tenomodulin (Abcam, TNMD, catalog # ab203676, 1:50) for tenogenesis evaluation; anti‐Collagen III (Abcam, COL III, catalog # ab6310, 1:100) for extracellular matrix (ECM) evaluation; anti‐CD86 (Abcam, catalog # ab53004, 1:100) and anti‐CD206 (Abcam, catalog # ab64693, 1:1000) for macrophage responses; anti‐CD31 (R&D, catalog # AF3628, 1:20), and anti‐alpha smooth muscle Actin antibody (Abcam, α‐SMA, catalog # ab7817, 1:200) for angiogenesis evaluation; anti‐Runt‐related transcription factor 2 (Abcam, RUNX2, catalog # ab76956, 1:100), and anti‐osteocalcin (Santa Cruz, OCN, catalog # sc365797, 1:50) for osteogenesis evaluation. Secondary antibodies included goat anti‐mouse IgG (H&L) (Abcam, catalog # ab6789, 1:500), goat anti‐rabbit IgG (H&L) (Abcam, catalog # ab205718, 1:500), donkey anti‐goat IgG (H&L) (Invitrogen, catalog # PA1‐28659, 1:500), donkey anti‐goat IgG (H&L) (Invitrogen, catalog # A21432, Alexa Fluor 555, 1:200), donkey anti‐mouse IgG (H&L) (Invitrogen, catalog # A21202, Alexa Fluor 488, 1:200), donkey anti‐rabbit IgG (H&L) (Invitrogen, catalog # A21206, Alexa Fluor 488, 1:200). Detailed information of all antibodies can be found in Table S1. Samples were deparaffinized and then processed for heat‐induced antigen retrieval using citrate antigen retrieval solution. Thereafter, samples were treated with a blocking solution (5% bovine serum albumin, catalog # 11021037, Thermo Fisher) and subsequently incubated with primary antibody overnight at 4°C followed by double‐distilled water wash. Then samples were incubated with secondary antibody for 1 h at room temperature. For immunohistochemistry (IHC) staining, the samples were incubated with the solutions in the Diaminobenzidine kit (Pierce™ DAB Substrate Kit, catalog # 34002, Thermo Fisher) for 1 min and then suspended in double‐distilled water. The samples were followed with hematoxylin stain and rinsed with double‐distilled water. For immunofluorescence (IF) staining, DAPI (DAPI Fluoromount‐G®, Birmingham, USA) was used as a nuclear counterstain. All images were acquired with the Leica DM5500 B microscope equipped with epifluorescence optics (Leica Microsystems, Wetzlar, Germany).

### 2.5 Histological evaluation scoring, anisotropy assessment, and semi‐quantification analysis of positive‐stained area

General histological examination was performed with H&E stained images. Three animal samples in each group were collected for analysis. The histological evaluation score system, including four parameters: (1) fiber structure, (2) fiber arrangement, (3) nuclei roundness, and (4) cell density, was modified from Movin's grading.^33^ We quantified all variables using a 0–3 scale, with 0 being normal and 3 being maximally abnormal (Table S2).^34^, ^35^ The region of interest (ROI) referred to the original tendon injury site containing the newly formed granulation tissue between the two surgical repair margins of Achilles tendon. Five sections were randomly selected and analyzed from each sample, and all scoring procedures were conducted blindly by three independent assessors and the averaged scores were used for analysis. Regarding the fiber anisotropy assessment, FibrilTool, which is an ImageJ plug‐in that support quantification of fibrillar structures in microscopy images,^36^ was used. The detailed protocol can be found in the literature.^36^ Besides, two ROI were set at the area of newly formed granulation tissue and the area of tenotomy margin, respectively (Figure S7). The parameter, called anisotropy (a score between 0 and 1, 0 for no order (purely isotropic arrays) and 1 for perfectly ordered, that is, parallel fibrils (purely anisotropic arrays), was adopted to quantify the orientation and anisotropy of fibrillar structures. For the semi‐quantitative analysis of Masson's trichrome stain and IHC stain, Image J 1.52q software was used and the percentage of the positive area was calculated according to the previous studies.^37^, ^38^ The pixel intensity values for any color ranged from 0 to 255, wherein 0 represents the darkest shade of the color and 255 represents the lightest shade of the color as standard. The ROI was set at the area of newly formed granulation tissue and the positive areas were quantified from six randomly selected fields with a magnification of 200× within the ROI from each animal samples. Then the images were deconvoluted and only the Masson's stained fibrotic area or DAB (3,3'‐Diaminobenzidine) immunostained areas were shown. The threshold was adjusted to reduce background noise. The percentages of the immunostained‐positive area from the images were averaged. A schematic diagram of the semi‐quantitative analysis was represented in Figure S7.

### 2.6 Morphological characterization

To assess morphological changes, Achilles tendon length, cross‐sectional area (CSA), gastrocnemius length, and gastrocnemius weight were measured immediately after harvesting. Achilles tendon length was defined as the distance from the proximal calcaneus insertion to the most distal myotendinous junction.^39^, ^40^ Gastrocnemius length was defined as the distance from the most distal portion of the myotendinous junction to the femur insertion. Tissue net weight was measured using an analytical balance (catalog # NBL 214e, Nimbus Analytical Balances, USA).

### 2.7 Biomechanical testing

The mechanical properties of the Achilles tendons were tested on day 28. Calf muscle–Achilles tendon–calcaneal bone complex was harvested from the hindlimb. On the calf muscle side, the muscle tissue was scraped from the tendon tissue and attached to two sandpapers for fixation. On the calcaneus side, the bone block was mounted into a custom‐made polymethyl methacrylate (PMMA) block containing a hole for fixation. The intramuscular tendon fiber (inside of calf muscle) was fixed between two sheets of sandpaper to provide strong fixation. The samples were fitted into the mechanical test machine (A Hounsfield Test Machine; H25K‐S, Hounsfield Test Equipment LTD, Surrey, UK) with upper and lower clamps. After that, a tensile test was performed with a load cell of 445 N (100 lb) at a testing speed of 6 mm/min. Tensile ultimate load was recorded as the highest load when the reconstructed tissue failed, and tensile failure strain was defined as the moment at which tissue samples failed. Stiffness was calculated from the slope of the load–displacement curve. Tensile modulus was defined as the slope of the linear region of the stress–strain curve.

### 2.8 Statistical analysis

All data were obtained from at least three independent experiments and were presented as mean ± SD. All analyses were performed using SPSS software (Version 25, USA). One‐way ANOVA and two‐way ANOVA with post hoc Tukey's multiple comparisons were conducted. Statistical significance was set at *p* < 0.05.

## 3 RESULTS

### 3.1 BG/SA hydrogel enhanced cell infiltration and collagen deposition

**FIGURE 1:**
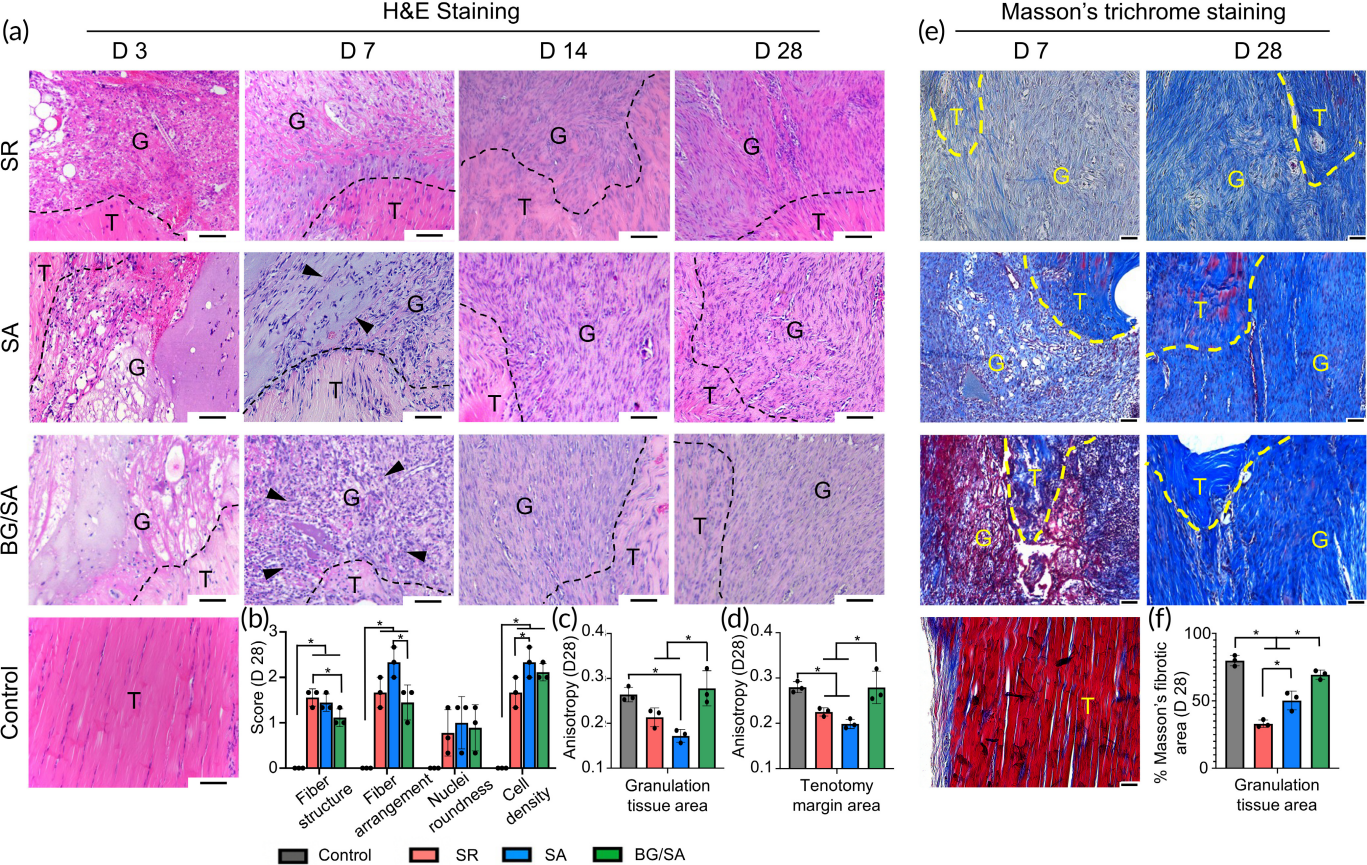
Histological analysis findings. (a) hematoxylin and eosin (H&E) staining of regenerated Achilles tendon in control, suture repair (SR), sodium alginate (SA), and bioactive glass (BG)/SA groups on Day 3, 7, 14, and 28 post‐surgery. Achilles tendon without injury serves as the control group. Black dotted lines indicate the tenotomy margin of the repair site. Black arrows denote cellular infiltration into the hydrogel. Scale bar, 100 μm. T, tendon tissue; G, granulation tissue. (b) Histological evaluation scores of tendon, in terms of fiber structure, fiber arrangement, nuclei roundness, and cell density, in control, SR, SA, and BG/SA groups on Day 28. (c) Degree of cell anisotropy in the granulation tissue area in control, SR, SA, and BG/SA groups on Day 28. (d) Degree of cell anisotropy in the tenotomy margin area in control, SR, SA, and BG/SA groups on Day 28. (e) Masson's trichrome staining of regenerated Achilles tendon in control, SR, SA, and BG/SA groups on Days 7 and 28 post‐surgery. Achilles tendon without injury serves as a control group. Blue: collagen fibers; red: cytoplasm; dark purple: nuclei. Yellow dotted lines indicate the tenotomy margin of the repair site. T, tendon tissue; G, granulation tissue. Scale bar, 50 μm. (f) Semi‐quantitative analysis of percentage of Masson's fibrotic area in control, SR, SA, and BG/SA groups on Day 28. Results for statistical analysis are presented as means ± SD; (*n* = 3; *p < 0.05).

The effect of BG/SA hydrogel on tendon healing was investigated using an in vivo model of rat Achilles tendon suture repair. H&E staining suggested that, on Day 7, there was increased cell density at the edge of the repair site in SA and BG/SA groups, while samples in BG/SA group exhibited a higher level of cell infiltration into the hydrogel around the edge of the repair site compared with the SA group (Figures 1a and S1). Moreover, enhanced cell alignment in granulation tissue was observed in BG/SA group on Days 14 and 28 when compared with that in SA and SR groups (Figure 1a). The whole tendon images on Day 28 are presented in Figure S2. In terms of histological scores evaluated on Day 28, BG/SA group outperformed SR group in the assessment of fiber structure and SA group in the assessment of fiber arrangement, respectively. (Figure 1b). Regarding the degree of cell anisotropy in newly formed granulation tissue and tenotomy area, BG/SA group exhibited increased anisotropy relative to SR and SA groups, the level of which was comparable to that of control group (Figure 1c,d). For Masson's trichrome staining, the control “physiologically normal” tendon appeared uniformly red, while the collagen in the granulation tissue appeared in blue, which indicated the presence of deposited collagen.^15^ When compared to SR group, on both Days 7 and 28, SA and BG/SA groups showed higher collagen deposition in the granulation tissue, evidenced by the high intensity of blue signal. The collagen fiber in BG/SA group appeared to disperse more uniformly, and the density of the collagen fiber was higher as compared with the fiber in the repair site treated with SA solution (Figure 1e). The semi‐quantitative analysis indicated an increased percentage of Masson's fibrotic area in BG/SA group relative to SR and SA groups (Figure 1f). However, no obvious difference in COL III expression can be observed among all experimental groups (Figure S3). All these results suggested that BG/SA hydrogel enhanced cell infiltration during the early phase and collagen deposition at a later time point during tendon healing.

### 3.2 BG/SA hydrogel dynamically regulated the macrophage phenotypic alteration during tendon healing

**FIGURE 2:**
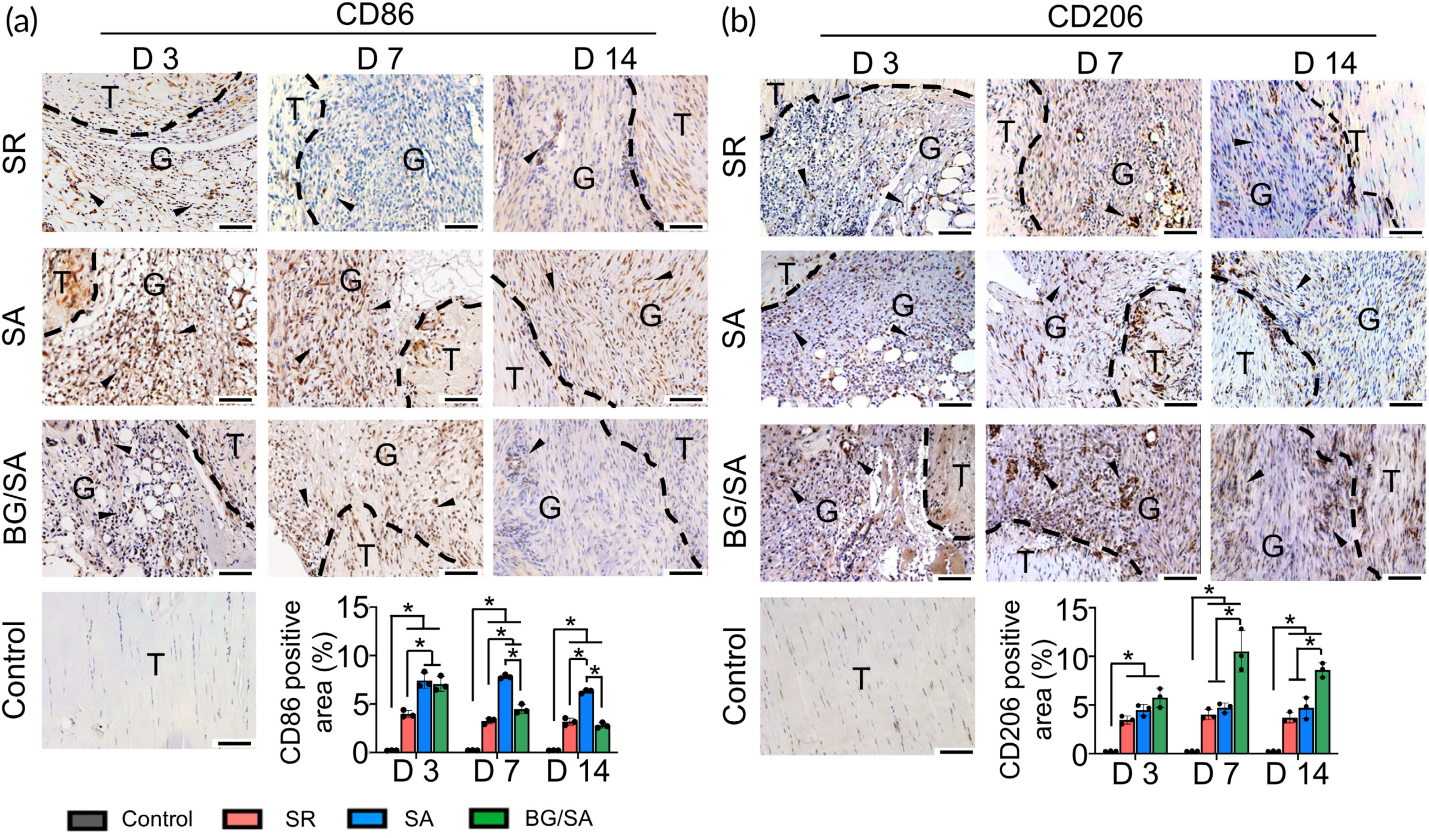
Immunohistochemistry (IHC) staining and semi‐quantitative analysis of CD86 and CD206 in regenerated Achilles tendon of control, suture repair (SR), sodium alginate (SA), and bioactive glass (BG)/SA groups on Days 3, 7, and 14 post‐surgery. (a) IHC staining and semi‐quantitative analysis of CD86 positive‐stained M1 macrophages in the granulation tissue in control, SR, SA, and BG/SA groups. (b) IHC staining and semi‐quantitative analysis of CD206 positive‐stained M2 macrophage macrophages in the granulation tissue in control, SR, SA, and BG/SA groups. Black dotted lines indicate the tenotomy margin of the repair site. Scale bar = 100 μm. T, tendon tissue; G, granulation tissue. Black arrows denote positive signals. Results for statistical analysis are presented as means ± SD; (*n* = 3; *p < 0.05).

The effect of BG/SA hydrogel on macrophage phenotype during tendon healing was investigated in vivo. Our data showed that more cells expressed CD86, a M1 macrophage marker, in granulation tissue in SA and BG/SA groups than SR group on Day 3, while BG/SA group exhibited less CD86 positive signals relative to SA group on Days 7 and 14. This phenomenon could be explained by the fact that the two hydrogel groups induced a more prominent acute M1 macrophage response for 3 days. Besides, the amount of M1 macrophages in the granulation tissue in BG/SA group was significantly less relative to SA group. These results implied that the increased accumulation of M1 macrophages was attributed to the presence SA, while the reduction of M1 macrophages in BG/SA group was likely the result of the presence BG (Figure 2a). On the other hand, the amount of CD206 positive‐stained cells, which represented M2 macrophages, was increased in BG/SA group relative to SR and SA groups from Day 3 to 7 and remained at a high level at Day 14. While the amount of CD86 positively stained cells in all experimental groups was gradually reduced from Days 3 to 14, the increased amount of M2 macrophages in BG/SA group suggested that BG, but not SA, enhanced the polarization of M2 macrophage in granulation tissue (Figure 2b). It is known that M1 and M2 macrophages sequentially contribute to different stages of tendon healing and the timing of the M1 to M2 phenotypic conversion acts as a critical determinant of the outcome of tissue regeneration.^41^ Therefore, our results showed that BG/SA hydrogel might improve tendon healing by regulating the phenotypic alteration of macrophages from M1 toward M2 subtypes.

### 3.3 BG/SA hydrogel improved angiogenesis during tendon healing

**FIGURE 3:**
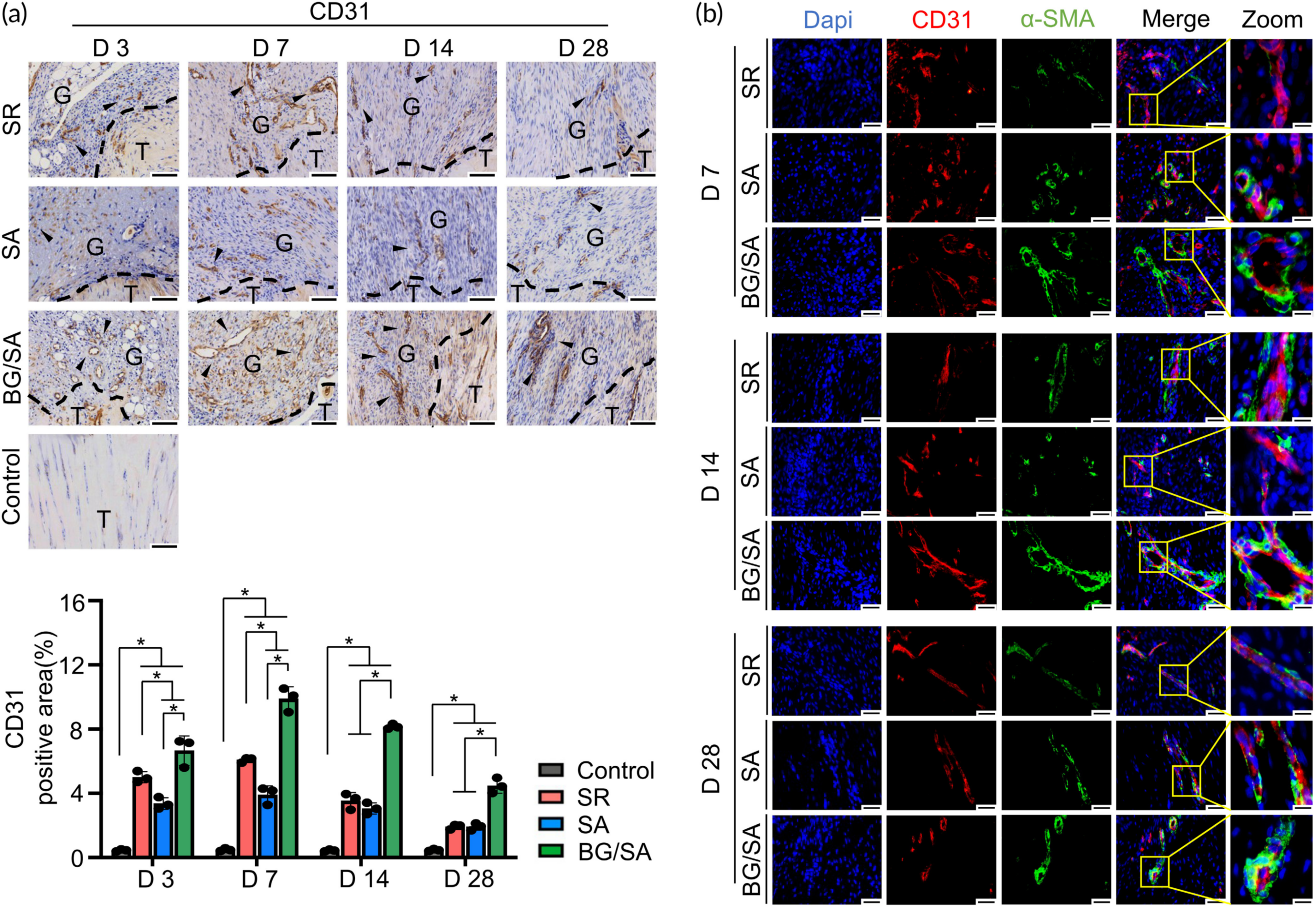
Immunostaining analysis of angiogenic markers. (a) Immunohistochemistry (IHC) staining and semi‐quantitative analysis of CD31 in regenerated Achilles tendon of control, suture repair (SR), sodium alginate (SA), and bioactive glass (BG)/SA groups on Days 3, 7, 14, and 28 post‐surgery. Scale bar = 100 μm. T, tendon tissue; G, granulation tissue. Black arrows denote positive signals. Results of statistical analysis are presented as means ± SD; (*n* = 3; *p < 0.05). (b) Immunofluorescence (IF) staining of CD31 and α‐SMA in regenerated Achilles tendon of SR, SA, and BG/SA groups on Days 7, 14, and 28 post‐surgery. Scale bar = 50 μm; Scale bar (zoom) = 20 μm

The IHC staining for CD31 and IF staining for CD31/α‐SMA were performed to investigate the angiogenesis in the granulation tissue during tendon healing. A larger amount of CD31 positive signals and CD31 positive ring‐like vascular structures were observed in the granulation tissue of BG/SA group, but not in the SA group, when compared with SR group from Days 3 to 28, indicating BG/SA hydrogel promoted the formation of vasculature (Figure 3a). Of note, the vascular network was not prevalent in the uninjured tendon site in both control and experimental groups, suggesting less demand of blood supply in a healthy tendon. We also found that the amount of CD31 positive signals were reduced on Day 28 when compared with that on Day 7 (Figure 3a). This phenomenon might attribute to the reduced requirement of nutrition and oxygen after successful regeneration, leading to the degeneration of capillaries.

Co‐stained IF images of CD31/α‐SMA were used for the further identification of the capillaries and mature blood vessels in the granulation tissue. Our results indicated that distinctly more co‐localization of CD31/α‐SMA positive signals could be observed in the granulation tissue of BG/SA and SA groups than those in the SR group on Days 7, 14, and 28, which indicated the formation of more mature blood vessels in the two hydrogel groups than in SR group. In line with IHC results, the size of positive co‐stained CD31/α‐SMA ring‐like vascular structures in the BG/SA group was larger than those in the SA group at all indicated time points (Figure 3b). Taken together, BG/SA hydrogel can significantly promote the formation of capillaries and mature blood vessels during tendon healing.

### 3.4 BG/SA hydrogel selectively regulated the spatial distribution of M2 macrophages and angiogenesis during tendon healing

**FIGURE 4:**
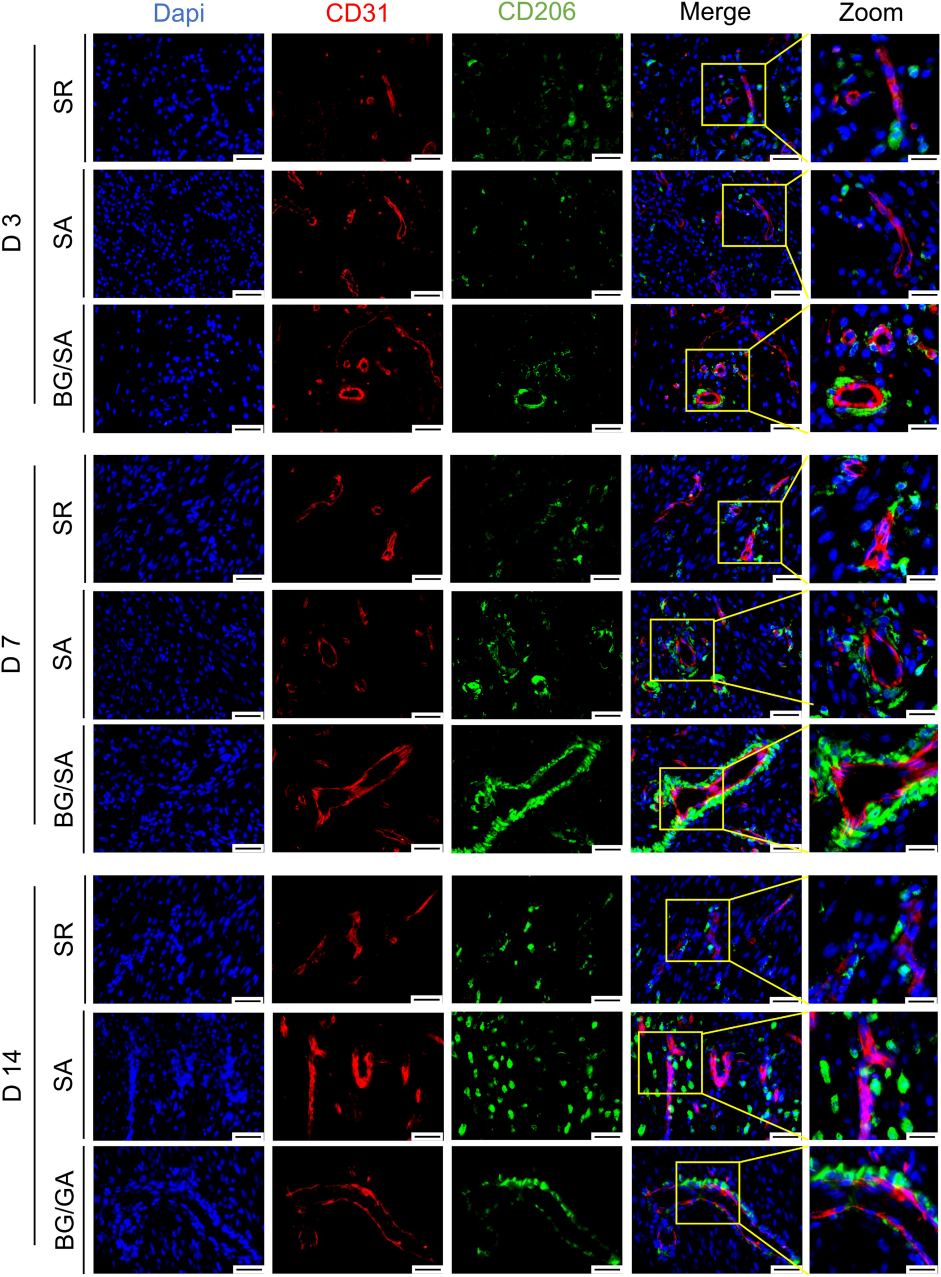
Immunofluorescence (IF) staining of CD31 and CD206 in regenerated Achilles tendon of suture repair (SR), sodium alginate (SA), and bioactive glass (BG)/SA groups. Scale bar = 50 μm; Scale bar (zoom) = 20 μm

As previously reported, M2 macrophage polarization is essential for promoting angiogenesis.^42^, ^43^ M2 macrophages release a number of pro‐angiogenic factors to stimulate angiogenesis and promote tissue regeneration.^44^ To identify the distribution of M2 macrophages and relationship between macrophage polarization and angiogenesis in the granulation tissue, IF staining was performed to co‐detect CD206 and CD31. Our results showed that, in BG/SA group, numerous CD206‐positive cells were located around the CD31‐positive vessels, most apparently on Day 7. However, this spatial distribution of M2 macrophages and vasculature was seldom observed in SR group throughout the experimental period (Figures 4 and S4). Therefore, our results revealed the association between the spatial accumulation of M2 macrophages in granulation tissue and the localization of CD31‐positive vessels, confirming the role of M2 macrophages in supporting angiogenesis.

### 3.5 BG/SA hydrogel accelerated tenogenesis without raising the risk of heterotopic ossification during tendon healing

**FIGURE 5:**
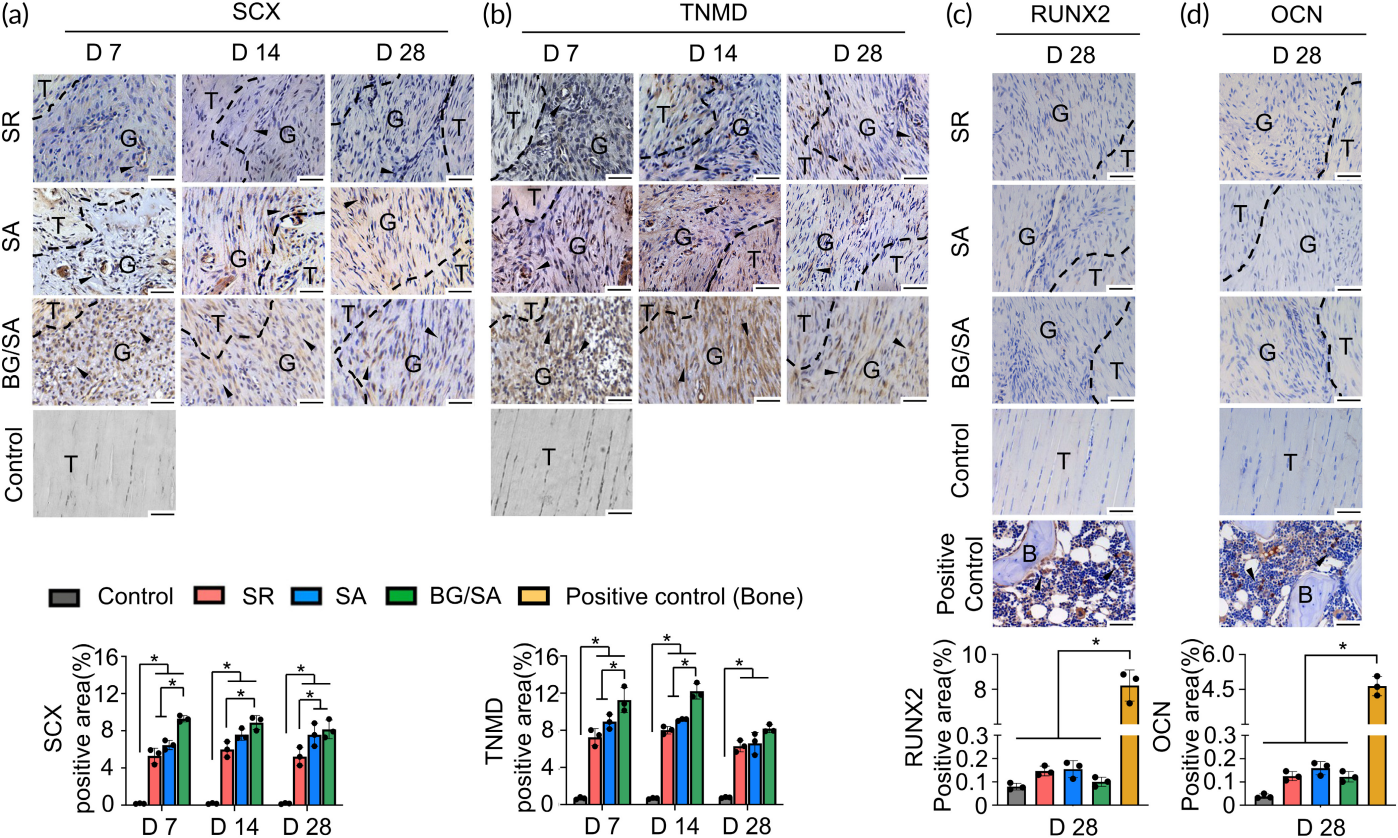
Bioactive glass (BG)/sodium alginate (SA) hydrogel promoted tenogenesis without raising the risk of heterotopic ossification in vivo. (a–d) Immunohistochemistry (IHC) staining and semi‐quantitative analysis of SCX (a), TNMD (b), RUNX2 (c), and OCN (d) during tendon healing. Rat knee joint samples serve as the positive control for RUNX2 and OCN staining. Black dotted lines indicate the tenotomy margin of the repair site. Scale bar = 50 μm. T, tendon tissue; G, granulation tissue; B, bone. Black arrows denote positive signals. Results for statistical analysis are presented as means ± SD; (*n* = 3; *p < 0.05).

The effect of BG/SA hydrogel on tenogenesis was investigated by performing IHC staining of tendon mature markers (SCX and TNMD). SCX is identified as a master regulator of embryonic tendon,^45^, ^46^ and TNMD is critical for tendon maturation and essential for the prevention of fibrovascular scar formation during early tendon healing.^47^ Our results showed that the levels of SCX and TNMD increased progressively in granulation tissue during tendon healing in all experimental groups. In particular, BG/SA group exhibited the highest levels of SCX and TNMD in granulation tissue at all time points (Figure 5a,b). Of note, the increased SCX and TNMD signals of BG/SA group on Day 7 might explain the high cell nuclei density (Figure 1a) and cytoplasm content (Figure 1b, red signal), which might be associated with the elevated fibroplasia.

Heterotopic ossification refers to the formation of pathological bone in soft tissue and can occur with soft tissue injury, including tendon injury.^48^ Given that BG has been reported to promote osteogenesis,^49^ it may, therefore, increase the risk of heterotopic ossification. To assess whether BG/SA hydrogel treatment induced heterotopic ossification, we performed IHC staining of RUNX2 and OCN (early and late osteolineage markers, indicating mineralization sites). According to our results, ectopic signals of RUNX2 and OCN were hardly detected in granulation tissue in all tendon groups on Day 28 (Figure 5c,d), suggesting a low risk of heterotopic ossification. In addition, we also performed Alizarin Red staining but failed to detect calcium deposition in the granulation tissue in all four groups (Figure S5). Taken together, BG/SA hydrogel accelerated tenogenesis without triggering ectopic bone formation in the tendon.

### 3.6 BG/SA hydrogel attenuated the pathological morphological changes of Achilles tendon and gastrocnemius muscle

**FIGURE 6:**
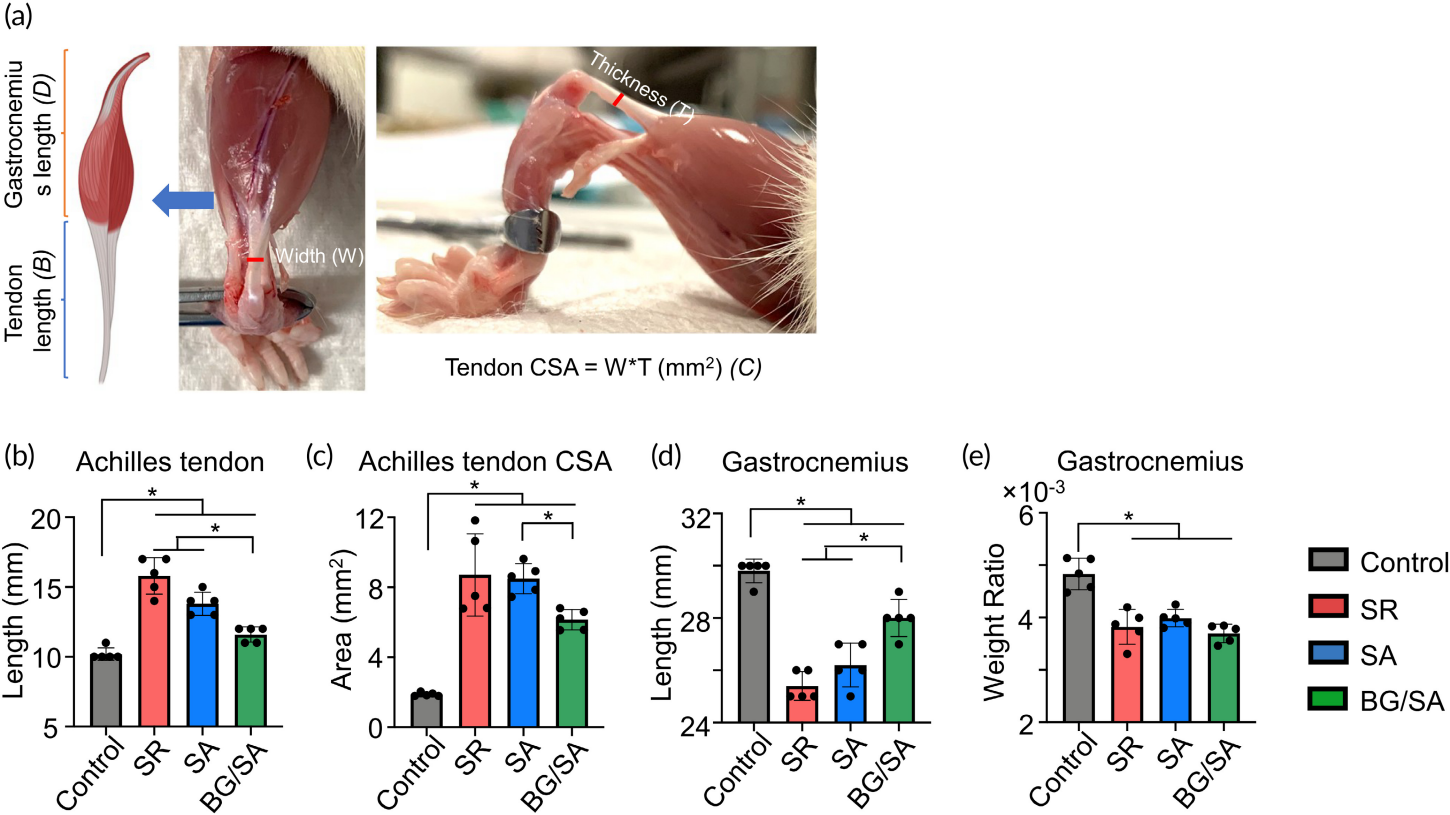
Bioactive glass (BG)/sodium alginate (SA) hydrogel selectively improved morphological properties of Achilles tendon and gastrocnemius muscle. (a) A schematic diagram and a picture of Achilles tendon and gastrocnemius muscle. (b,c) Length (b) and, cross‐sectional area (CSA) (c) of Achilles tendon on Day 28 post‐surgery. (d,e) Length (d) and mass (e) of gastrocnemius muscle were assessed on Day 28 post‐surgery. Results for statistical analysis are presented as means ± SD; (*n* = 5; *p < 0.05).

Tendon elongation and gastrocnemius muscle atrophy are functional deficits that can occur during tendon healing,^50^ which are displayed by an early increase of the tendon CSA in the proliferation phase, followed by decreased CSA during the remodeling phase.^51^, ^52^ Tissue morphological parameters, including tendon length, CSA, gastrocnemius muscle weight ratio, and gastrocnemius length, were measured on Day 28 to assess the effect of BG/SA hydrogel on suture repair of Achilles tendon (Figure 6a).

Our results suggested that SR group exhibited an increased Achilles tendon length compared with the control group, while BG/SA group, but not SA only, showed reduced Achilles tendon length compared with the SR group (Figure 6b). Besides, although the Achilles tendon CSA of SR, SA, and BG/SA was larger than that of control, the tendon CSA of BG/SA was significantly smaller than that of SA (Figure 6c). In addition, our results showed that tendon rupture‐induced gastrocnemius muscle atrophy did not improve by suture repair (SR group), whereas BG/SA treatment increased the gastrocnemius length compared with SR and SA groups (Figure 6d). There was no significant effect of BG/SA on gastrocnemius weight as compared with SR and SA groups (Figure 6e). Taken together, BG/SA hydrogel appeared to reduce detrimental morphological changes of the Achilles tendon and alleviate the deterioration of gastrocnemius muscle atrophy slightly.

### 3.7 BG/SA hydrogel improved biomechanical properties of calf muscle–Achilles tendon–calcaneal bone complex

**FIGURE 7:**
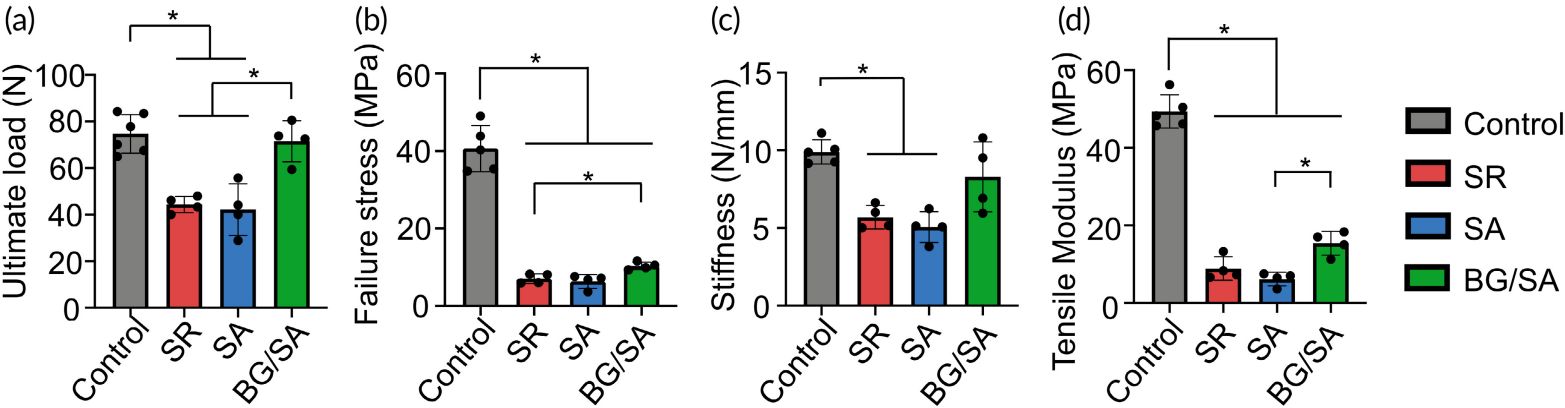
Biomechanical testing of Achilles tendon on Day 28 post‐surgery. (a–d) Ex vivo biomechanical testing. Ultimate load (a), failure stress (b), tendon stiffness (c), tensile modulus (d) of reconstructed tendons. Results for statistical analysis are presented as means ± SD; (*n* = 5 in control group, *n* = 4 in suture repair [SR], sodium alginate [SA], and bioactive glass [BG]/SA groups; *p < 0.05).

To assess the effect of BG/SA hydrogel on the biomechanical properties of the reconstructed tendons, we performed ex vivo biomechanical testing on the calf muscle–Achilles tendon–calcaneal bone complex on Day 28 postinjury. Compared with control, SR and SA did not improve ultimate load, while BG/SA significantly increased ultimate load toward the level of the control group (Figure 7a). BG/SA also increased failure stress as compared with SR group (Figure 7b). In addition, BG/SA group exhibited comparable stiffness relative to the control group (Figure 7c) and improved tensile modulus (Figure 7d) relative to the SA group. The representative stress–strain curves, schematic and pictures of actual experimental setup for biomechanical tests are presented in Figure S6. Overall, our results suggested that BG/SA hydrogel could improve the tensile properties of the reconstructed calf muscle–Achilles tendon–calcaneal bone complex.

## 4 DISCUSSION

Tendon rupture remains a significant clinical challenge due to the poor patient response to current treatments that result in an exhausting and lengthy rehabilitative process and impaired quality of life. In this study, we developed an injectable BG/SA hydrogel‐based therapy to facilitate tendon healing. Our results indicated that injectable BG/SA hydrogel, with immunomodulatory and angiogenic properties, promoted tenogenesis, and enhanced the tendon biomechanical properties without increasing the risk of heterotopic ossification at the tendon healing site.

BG has been recognized as one of the promising biomaterials for regenerative medicine, especially for tissue healing applications, due to its ability to shorten healing time.^53^, ^54^ Notably, the ability of BG to regulate phenotypic changes of macrophages and to promote the formation and maturation of blood vessels has been considered the major benefit in the case of soft tissue healing.^15^, ^55^ Specifically, it has been proved that BG stimulated M2 macrophages to release anti‐inflammatory cytokines and angiogenic factors, such as VEGF, TGF‐β, and FGF2, thus attracting fibroblasts and endothelial cells for provisional granulation tissue formation and angiogenesis.^19^ In addition to the skin wound healing,^15^ BG has also been proposed for regenerating other soft tissue, such as heart and lung.^56^ Specifically, enhancing fibroblast proliferation, inducing angiogenesis, and eliciting antibacterial activity were supported by introducing BG.^57^ Besides, a previous study reported that BG can improve the integration between tendon and bone in rotator cuff tendon repair.^58^ Therefore, we speculated that BG may contribute to tendon tissue healing, and our results demonstrated that delivering BG via an injectable hydrogel could be a promising therapeutic option.

Innate immune rejection is a major issue following biomaterials implantation as it compromises tissue healing in vivo. Developing biomaterials capable of modulating macrophage polarization could improve the success rate of biomaterial‐based approaches for regenerative medicine. The timely modulation of macrophage phenotype appears to be critical during the host tissue response following biomaterial administration and the associated remodeling process at the implantation site.^12^ Macrophages, among other immune cells that are present during the host tissue response, have remarkable plasticity to respond to environmental changes to exert different functions, thus contributing to different stages of tissue healing.^59^, ^60^ Consequently, depletion of macrophages has been shown to abolish the healing process completely.^61^ Recently, a systematic review reported that macrophage polarization might be associated with the extent of tendon healing and proposed that modulation of macrophage phenotype can lead to potential novel therapeutic options.^62^ A previous study found that stem cell exosome‐treated macrophages, which presented a M2 phenotype, were able ot promote early Achilles tendon healing.^32^

Tendon is poorly vascularized and heals poorly with the formation of fibrous tissue and scar tissue compared to other musculoskeletal tissues.^10^ The connective scar tissue often signifies inferior functional properties at the injury site.^63^ Angiogenesis, the process of new blood vessel formation from existing blood vessels, is a key aspect of virtually all repair processes.^64^ Although the role of angiogenesis in tendon repair remains controversial given that chronic tendinopathy exhibits neovascularization that is associated with innervation and pain, previous studies have reported that activation of angiogenesis during the early stage of tendon healing could promote tenogenesis.^65^ Several studies demonstrate that a subtype of pro‐angiogenic macrophages exhibits an M2 phenotype,^66^, ^67^ and M2 macrophages not only facilitate the differentiation of endothelial progenitor cells^68^, ^69^ but also influence all stages of angiogenesis via release of various growth factors, including VEGF‐A and VEGF‐C.^70^ VEGF is an endogenous stimulator of both angiogenesis and increased vascular permeability that is believed to be essential for neovascularization.^71^, ^72^ Therefore, the enhanced pro‐angiogenic activity could be contributed by polarized M2 macrophages, which served as supporting cells to guide the fusion of endothelial tip cells and facilitate vascular sprouting.^73^ Our results demonstrated that BG/SA hydrogel could stimulate tendon healing in the increased presence of M2 macrophages compared with the current surgical treatment in a rat Achilles tendon repair model. We also revealed the spatial and temporal association between M2 macrophages and endothelial cells, providing evidence about the relationship between the polarization state of macrophages and the progressive stages of angiogenesis. This is supported by results of a previous in vitro study, which reported that BG ionic products stimulated M2 polarization of macrophages in vitro and subsequently enhanced the vascularization of endothelial cells and the synthesis of ECM protein by fibroblasts.^19^ Based on the secretion profile of different growth factors by M1 and M2 macrophages and their apparent synergistic behaviors in angiogenesis, Spiller et al. concluded that M1 macrophages stimulated capillary sprouting, while M2 macrophages aided vessel stabilization through pericyte recruitment.^74^ This further supported the idea of modulating macrophage phenotype as potential novel therapeutic options for tendon healing.

For evaluating the extent of tendon healing, multifaceted assessments, including histological and morphological characterization, biomechanical testing, and analysis of tenogenesis were performed. In our study, BG/SA hydrogel was shown to accelerate tenogenesis, partially revert pathological changes of Achilles tendon after injury, and improve tensile properties of the reconstructed tendon tissue. Upon reviewing literature, we could conclude that our results were comparable to or even superior to the outcomes reported in some previous studies. For example, Muller et al. reported that the administration of collagen type I sponge in rat Achilles tendon defect improved ultimate load but resulted in a higher CSA compared with the control.^75^ By applying adipose‐derived stem cell exosomes on injured patellar tendon, Liu et al. reported a similar tenogenesis outcome to ours, in which the tenogenesis markers, including TNMD and SCX, were detected at a higher expression level from 2 to 4 weeks post‐surgery relative to the control groups.^76^ Chamberlain et al. performed the injection of mesenchymal stem cell‐derived extracellular vesicle‐induced macrophages into the injured site of Achilles tendon and showed that only the tensile modulus exhibited a significant increase on Day 14 post‐surgery, whereas there was no significant difference in ultimate load and stiffness relative to control group.^32^

Although our results suggested that BG/SA could be a promising treatment option, the risk of ossification should be evaluated when applying BG in soft tissue healing given that BG exhibits osteogenic property.^49^ Our approach of administering an injectable BG/SA hydrogel ensured BG was delivered to the tendon repair site locally. Besides, the presence of osteoprogenitor and osteoblast markers was not detected at the repair site, suggesting our treatment did not induce heterotopic ossification. Despite BG's known potential to stimulate bone regeneration, it has been tested in regenerating soft tissues such as skin and heart.^15^, ^56^ Similar to our report, these studies also did not report the occurrence of heterotopic ossification after the administration of BG. Our speculation of the reason behind is that an osteogenic‐promoting microenvironment, composed of insoluble extracellular matrix, biological cues (e.g., FGF‐2), osteoprogenitor cells and other factors, is needed to promote efficient osteogenesis.^77^ Tendon tissue, like other soft tissues such as skin and heart, may not provide such an optimal osteogenic‐promoting environment to stimulate bone formation. A previous study showed that tendon‐derived decellularized matrix could promote tenogenesis of tendon progenitor cells, while bone‐derived decellularized matrix promoted their osteogenic differentiation.^78^ In our current study, we focused on creating an injury in the tendon tissue only, thus there would be no exposure to the bone extracellular matrix or osteoprogenitor cells, including bone marrow stem cells, from the calcaneus bone insertion or other cortical bone sites. This might explain the minimal risk of heterotopic ossification observed in our study.

**FIGURE 8:**
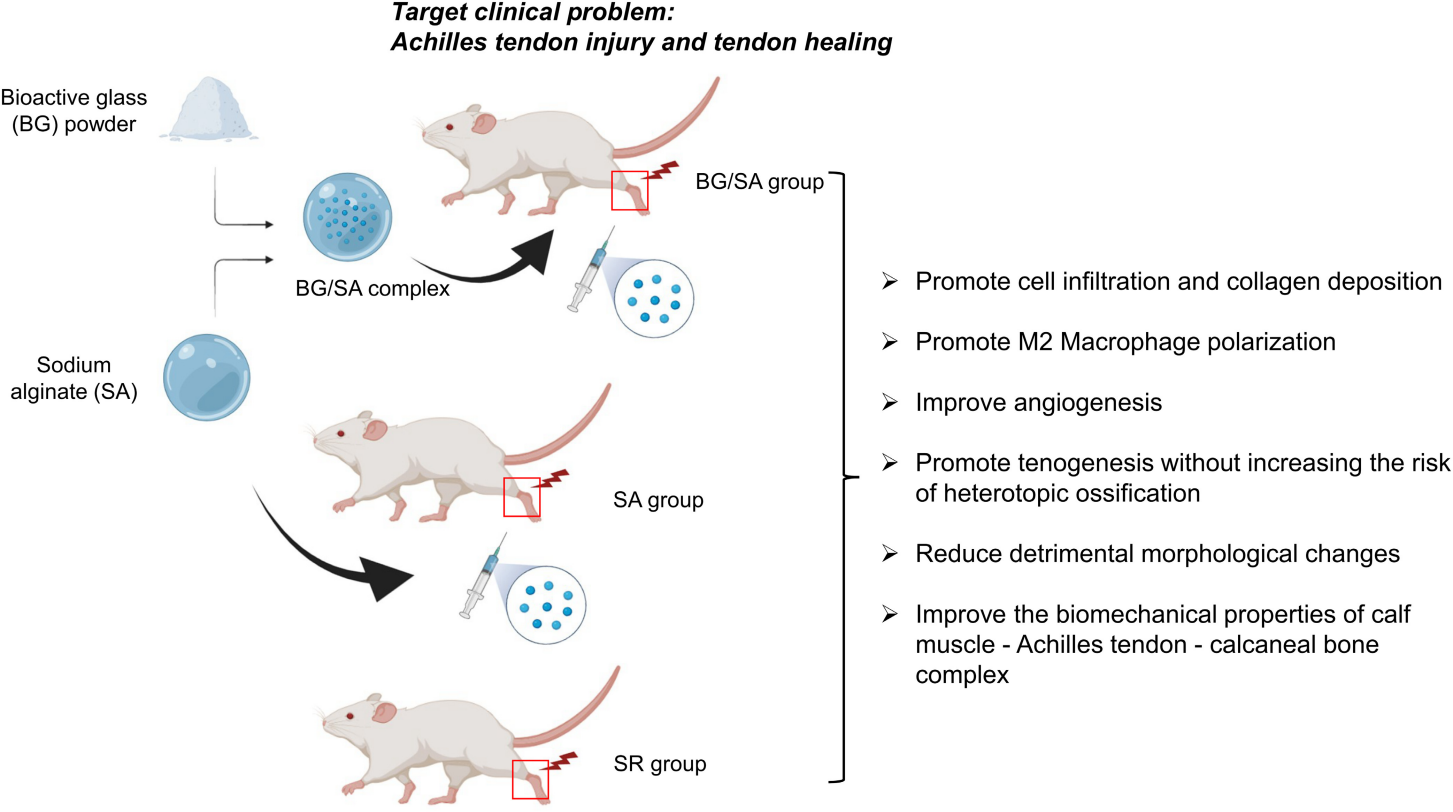
Schematic diagram of overall experimental design and major findings

The use of injectable BG/SA hydrogel in Achilles tendon surgical treatment also has a number of advantages (Figure 8). First, BG/SA hydrogel is developed from clinically approved biomaterials that should facilitate clinical translation and regulatory approval. Second, BG/SA hydrogel can be injected directly into the defect site or suture site where it will form a hydrogel to deliver BG to the tendon repair area directly and prevent BG diffusion into the surrounding tissue. Finally, delivering BG/SA hydrogel is a promising therapeutic approach for soft tissue healing without the risk of heterotopic ossification.

There were some limitations in our study. First, in the present study, the tendon injury was created by a surgical blade at the mid‐substance of Achilles tendon, the most widely used method in tendon rupture‐related studies. Such a cutting injury model differs from one in patients whose Achilles tendon usually suffers from a high‐energy traumatic rupture. Second, although improvement in tendon regeneration was observed in BG/SA group, only a single dose of BG/SA was applied for implantation. Therefore, we will consider establishing a stripped tendon rupture model and administering multiple doses of the BG/SA hydrogel in our future studies.

## 5 CONCLUSIONS

This is the first study investigating the therapeutic effect of an injectable BG/SA hydrogel on accelerating tendon healing. Histological, morphological, and biomechanical analyses demonstrated that BG/SA hydrogel accelerated tenogenesis, partially reverted pathological changes of Achilles tendon after injury, and improved tensile properties of the reconstructed tendon tissue. Injectable BG/SA hydrogel, with immunomodulatory and angiogenic properties, is a novel, promising and practical therapeutic option to augment tendon healing in conjunction with surgical intervention in a clinical setting.

## AUTHOR CONTRIBUTIONS

**Hongtao Xu:** Conceptualization (equal); data curation (lead); formal analysis (lead); investigation (lead); methodology (equal); resources (equal); writing – original draft (equal). **Yanlun Zhu:** Conceptualization (equal); investigation (lead); methodology (equal); resources (equal); writing – original draft (equal). **Jiankun Xu:** Formal analysis (supporting); validation (supporting); writing – review and editing (supporting). **Wenxue Tong:** Resources (supporting); validation (supporting). **Shiwen Hu:** Data curation (supporting); resources (supporting). **Yi‐Fan Chen:** Validation (supporting). **Shuai Deng:** Validation (supporting). **Hao Yao:** Validation (supporting). **Jie Li:** Validation (supporting). **Hon Fai Chan:** Conceptualization (equal); funding acquisition (equal); investigation (equal); methodology (equal); project administration (equal); supervision (equal); writing – review and editing (equal).

## CONFLICT OF INTEREST

The authors have no conflicts of interest to declare.

### PEER REVIEW

The peer review history for this article is available at https://publons.com/publon/10.1002/btm2.10345.

## Supporting information


**Appendix S1** Supporting Information

## Data Availability

The data that support the findings of this study are available from the corresponding author upon reasonable request.
