# Supplementary material for: Injectable bioactive glass/sodium alginate hydrogel with immunomodulatory and angiogenic properties for enhanced tendon healing
Source: Bioeng Transl Med. 2022 Jun 3;8(1):e10345. doi: 10.1002/btm2.10345 (PMC9842034; doi:10.1002/btm2.10345)

**SUPPLEMENTARY DATA**

**Injectable Bioactive Glass/Sodium Alginate Hydrogel with Immunomodulatory and Angiogenic Properties for Enhanced Tendon Healing**

Hongtao XU^#1,2,3^, Yanlun ZHU^#4,5^, Jiankun XU^1,2^, Wenxue Tong^1,2^, Shiwen HU^1,2^, Yi-Fan Chen^6,7,8,9^, Shuai DENG^4,5^, Hao YAO^1,2^, Jie LI^10^, Chien-Wei LEE^*11^, Hon Fai CHAN^*4,5,12,13^

# Hongtao XU and Yanlun ZHU contributed equally to this study.


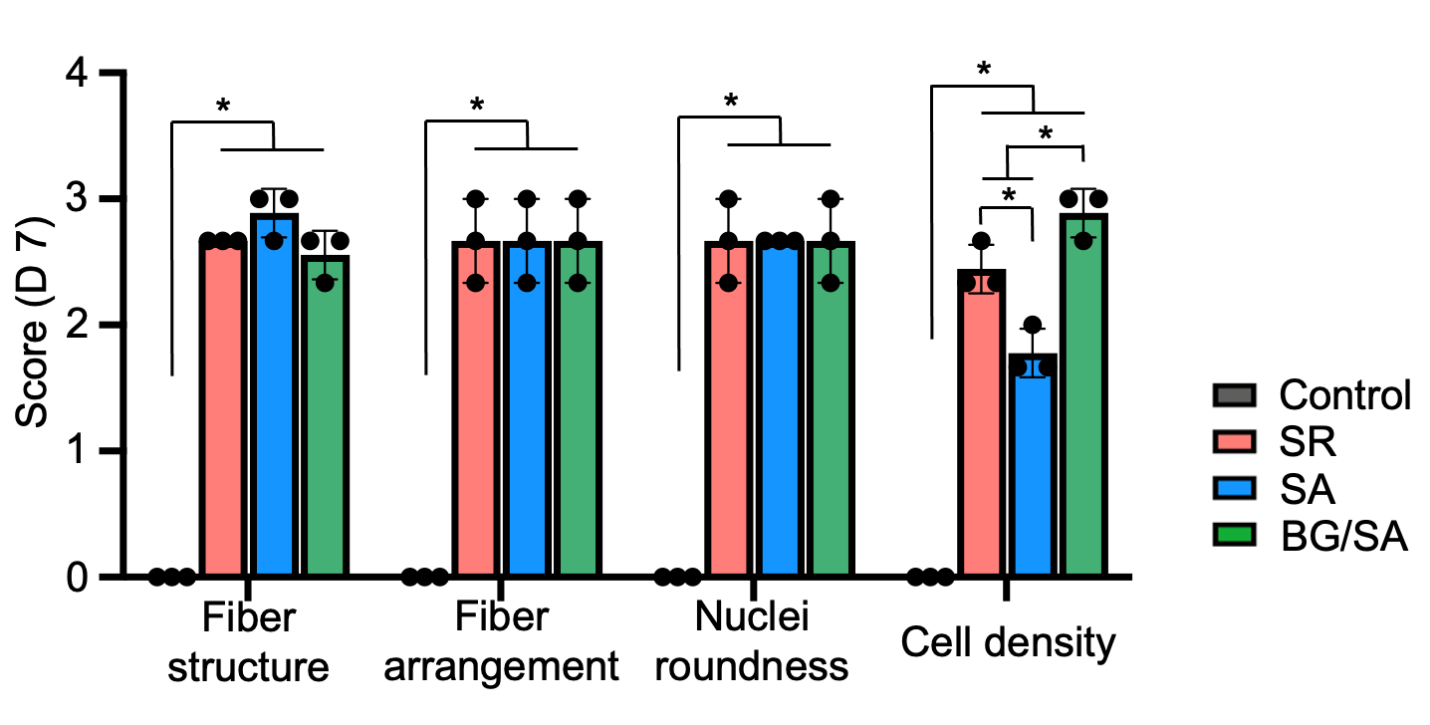


**Figure S1.** Histological evaluation scores of regenerated tendon, in terms of fiber structure, fiber arrangement, nuclei roundness, and cell density, in Control, SR, SA, and BG/SA groups on day 7. Results for statistical analysis are presented as means ± SD. (n = 3; *, P < 0.05).


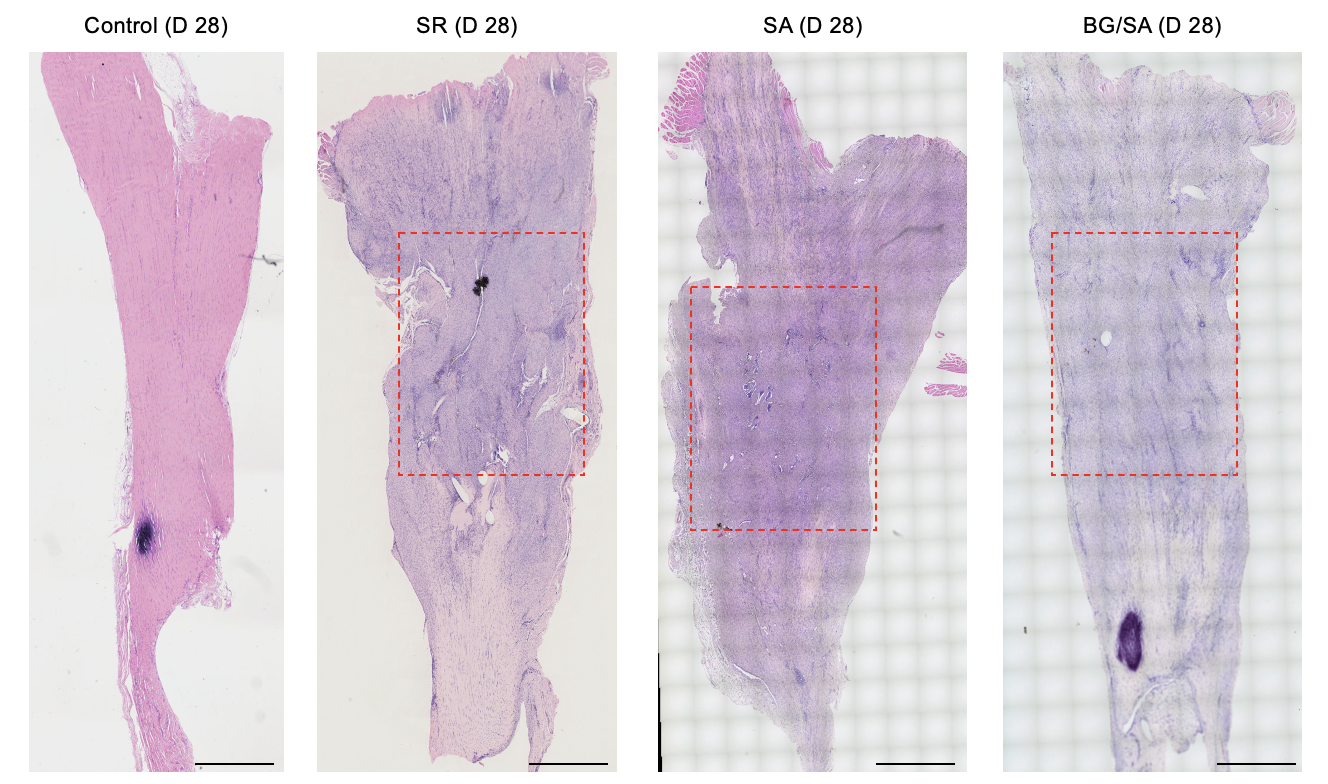


**Figure S2.** H&E staining of whole Achilles tendon of Control, SR, SA, and BG/SA groups on day 28 post-surgery. Area enclosed by the red dotted line denotes the granulation tissue. Scale bar = 1000 μm.


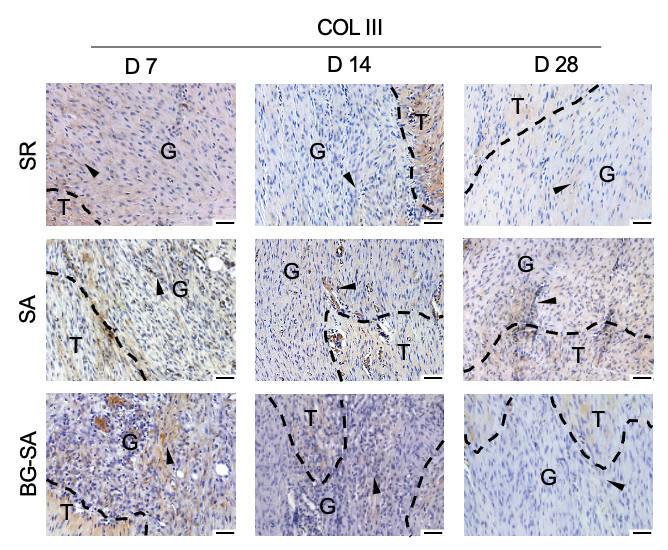


**Figure S3.** IHC staining of COL III in regenerated Achilles tendon of SR, SA, and BG/SA groups on days 7, 14, 28 post-surgery. Black dotted lines indicate the margins of the repair site. Black arrows indicate positive signals. Scale bar = 50 μm. T: tendon tissue; G: granulation.


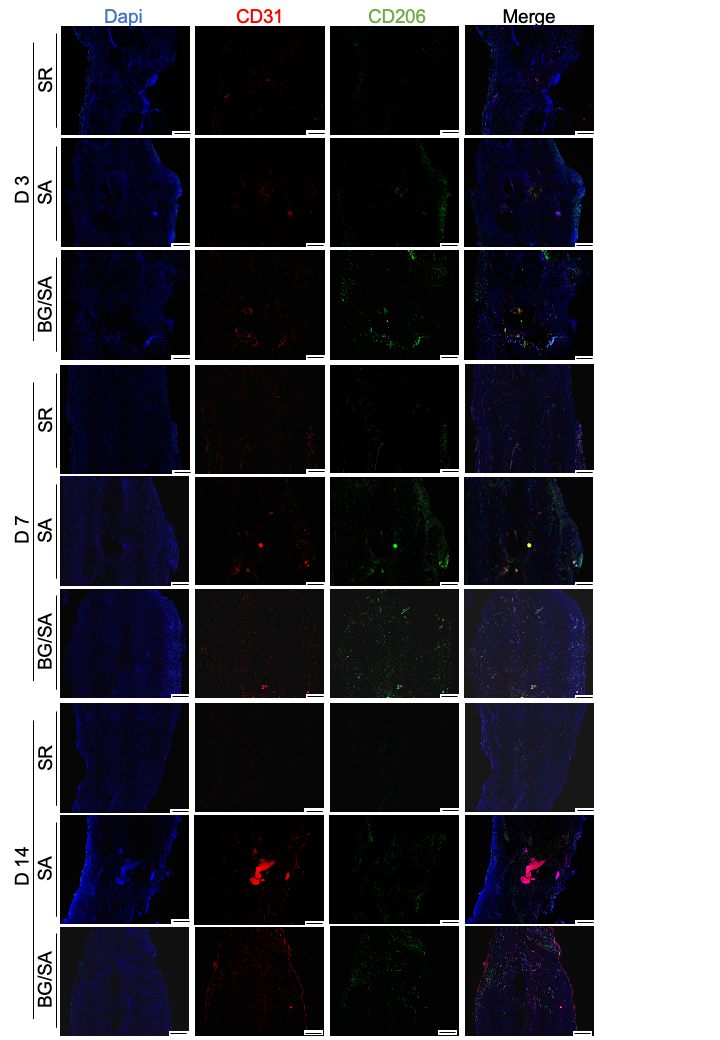


**Figure S4.** IF staining of CD31 and CD206 in regenerated Achilles tendon of SR, SA, and BG/SA groups on days 3, 7, 14 post-surgery. Scale bar = 1000 μm.


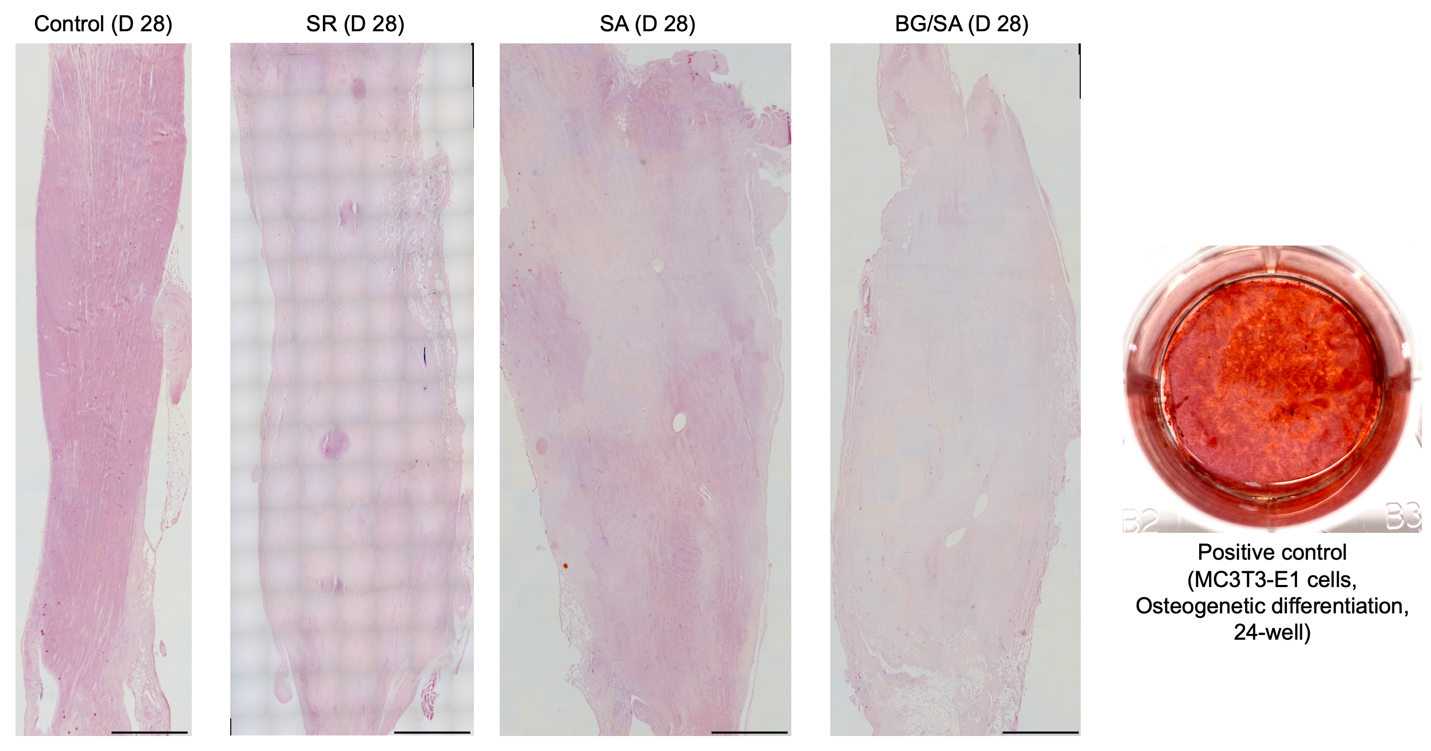


**Figure S5.** Alizarin Red staining of Control, SR, SA, and BG/SA groups on day 28 post-surgery.


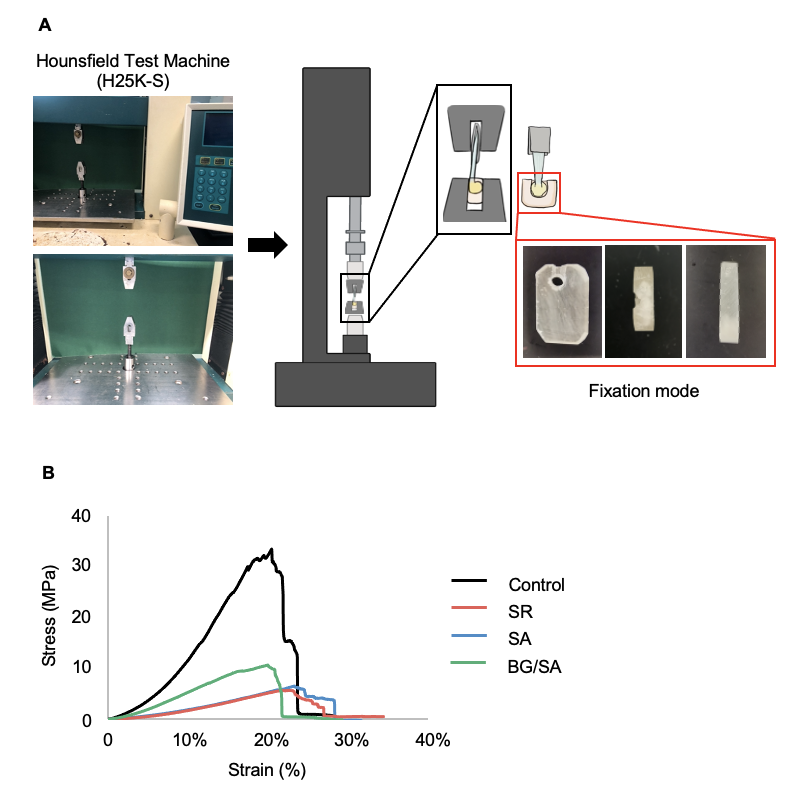


**Figure S6.** A schematic diagram of biomechanical fixation mode and representative stress-strain curves of Control, SR, SA, and BG/SA groups.


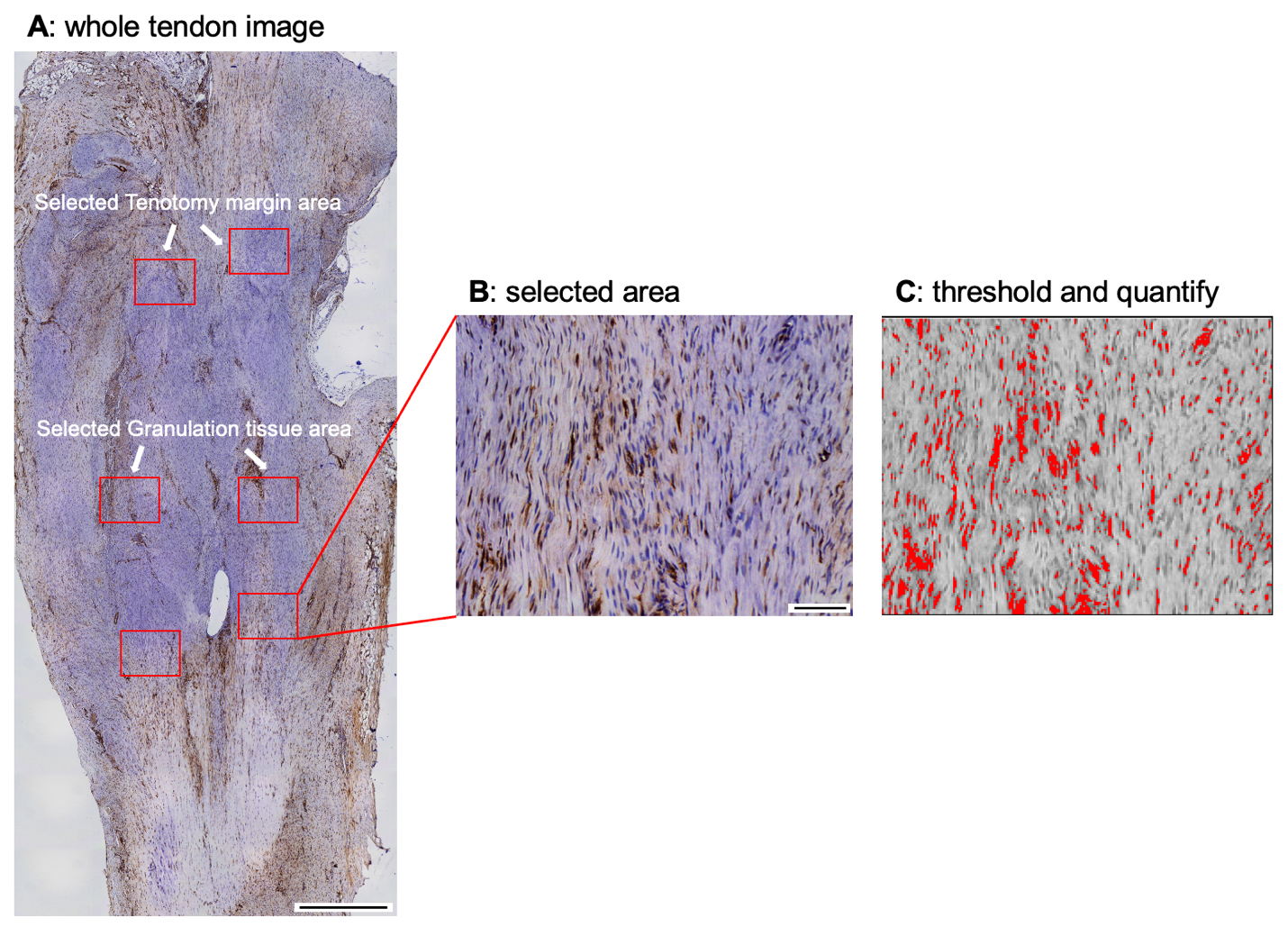


**Figure S7.** A schematic diagram of semi-quantitative analysis. (A) Scale bar = 1000 μm. (B) Scale bar = 100 μm

**Table S1.** List of all antibodies


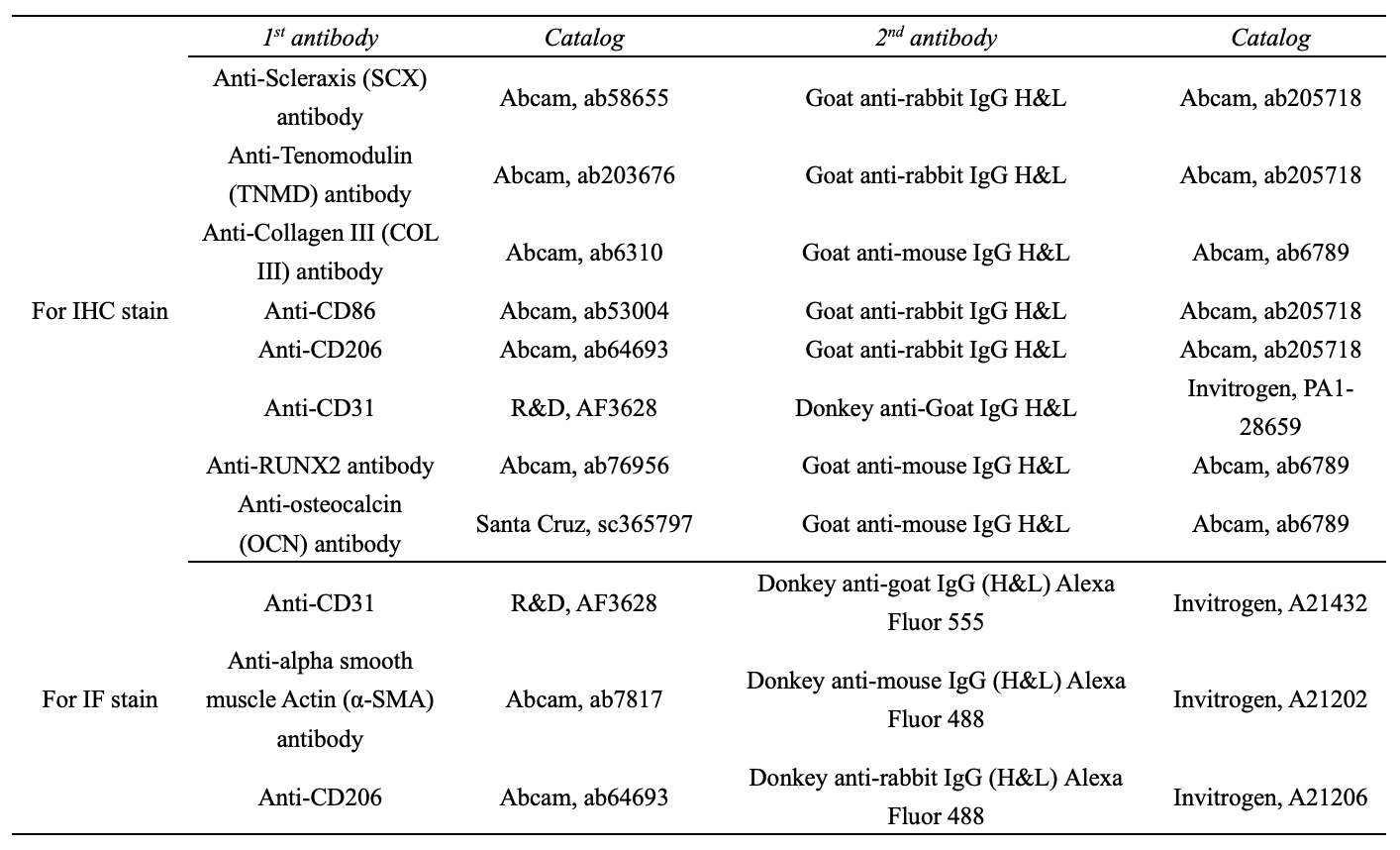


**Table S2.** Histological evaluation scores of tendon


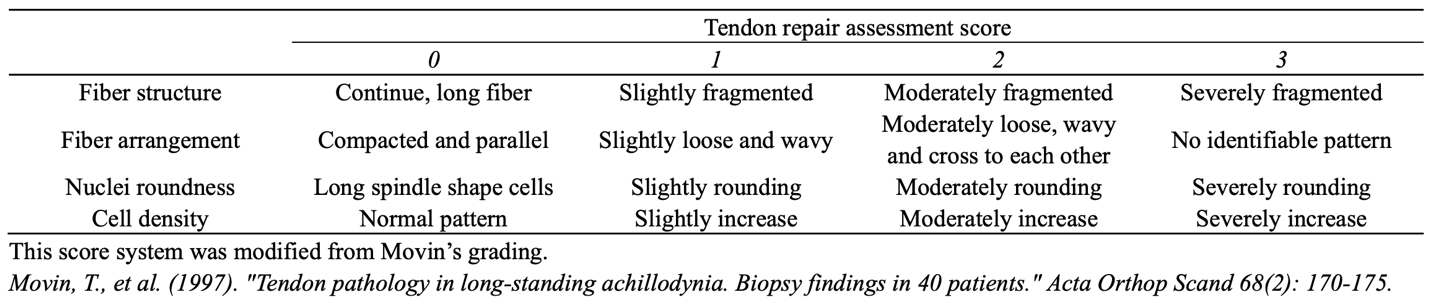

Supplement: Supplementary file 1 — Appendix S1 Supporting Information [file BTM2-8-e10345-s001.docx]
